# Supplementary figures and images for: Priming of leaf litter decomposition by algae seems of minor importance in natural streams during autumn
Source: PLoS One. 2018 Sep 7;13(9):e0200180. doi: 10.1371/journal.pone.0200180 (PMC6128472; doi:10.1371/journal.pone.0200180)

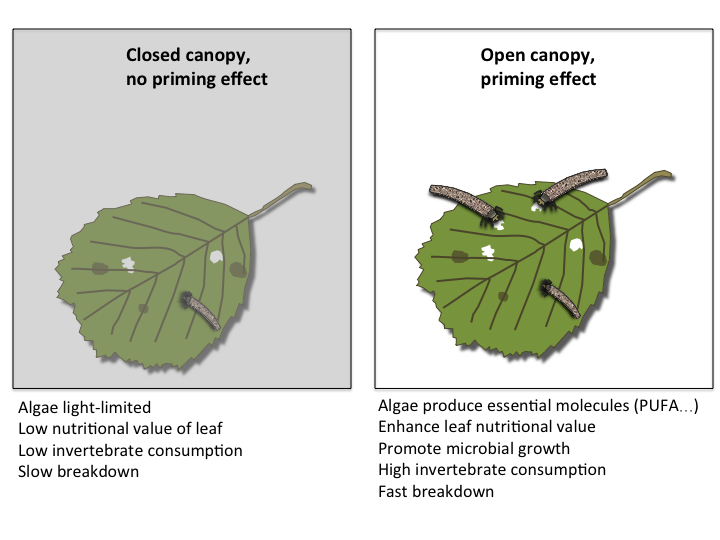

Supplement: S1 Fig — (TIFF) [file pone.0200180.s001.tiff]

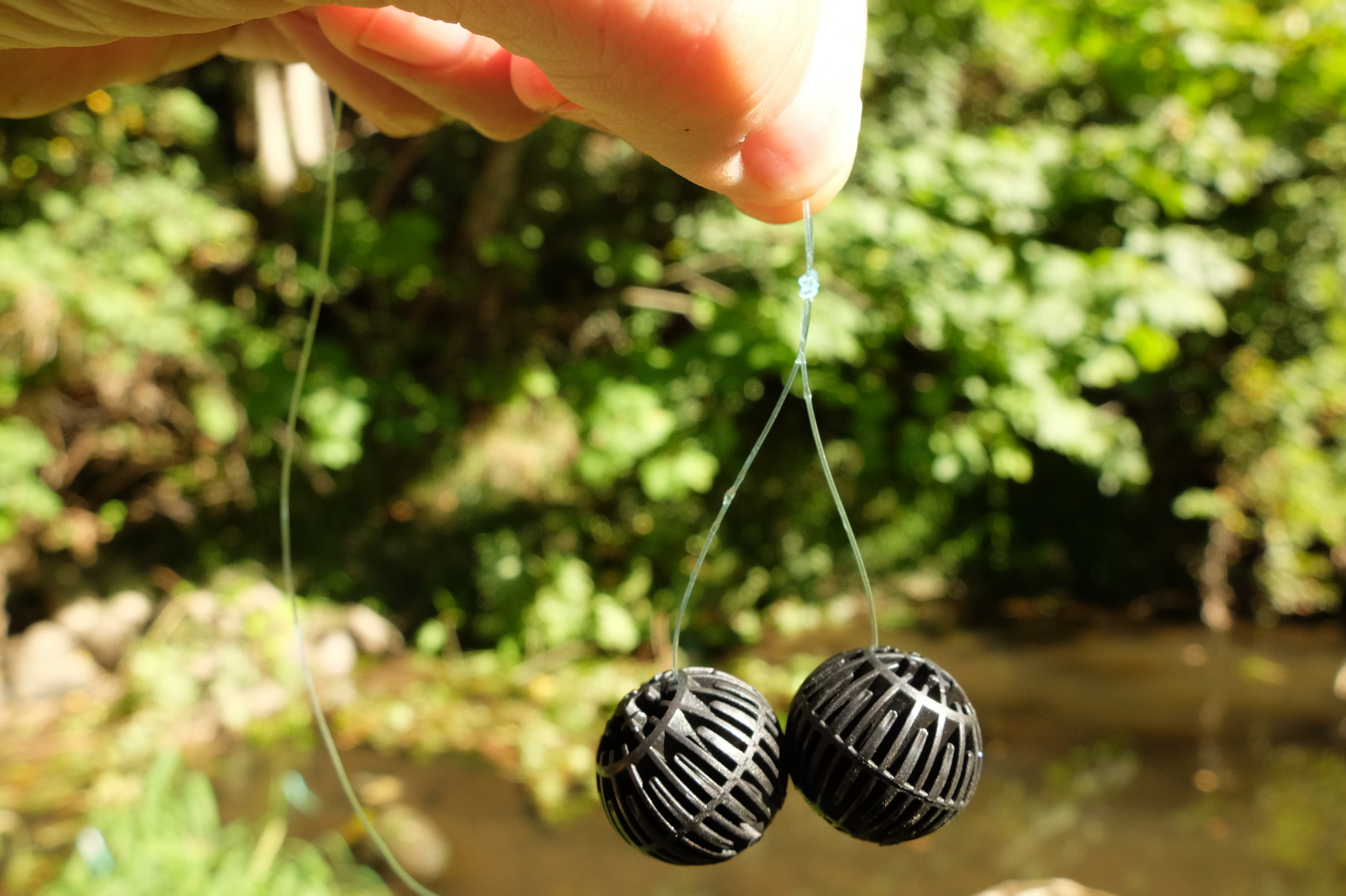

Supplement: S2 Fig — (TIFF) [file pone.0200180.s002.tiff]

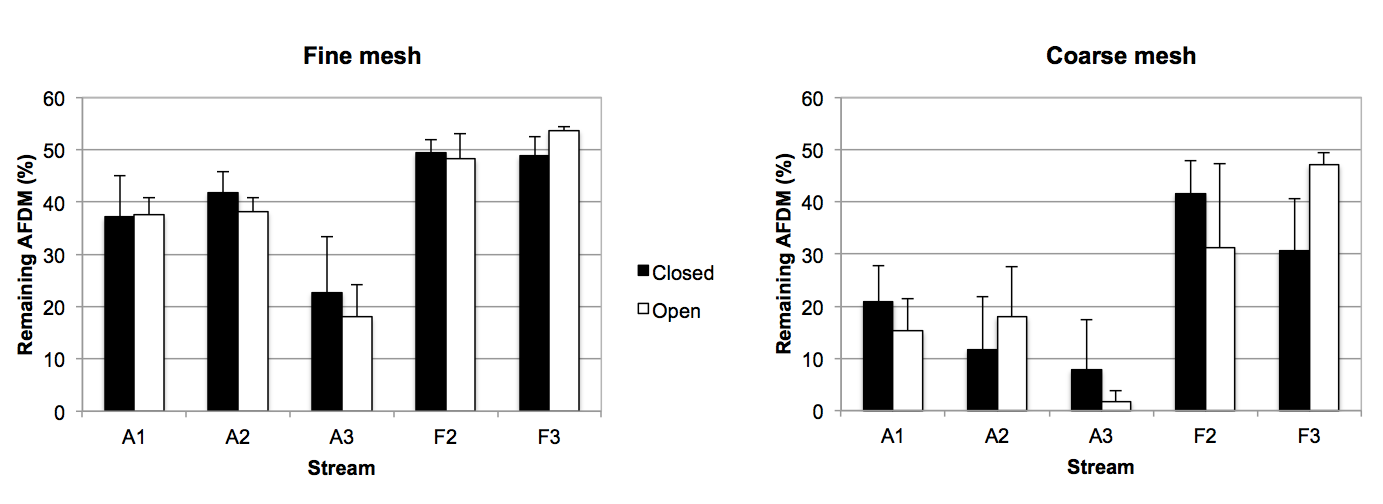

Supplement: S3 Fig — Leaf AFDM remaining in fine- (left) and coarse-mesh bags (right) by the end of the experiment in agricultural (A) and forest (F) streams. (TIFF) [file pone.0200180.s003.tiff]
